# A fixed inhaled nitrous oxide/oxygen mixture as an analgesic for adult cancer patients with breakthrough pain: study protocol for a randomized controlled trial

**DOI:** 10.1186/s13063-016-1739-9

**Published:** 2017-01-11

**Authors:** Qiang Liu, Yu Wang, Xiang-Jiang Luo, Ning-Ju Wang, Ping Chen, Xin Jin, Guo-Xia Mu, Xiao-Min Chai, Yue-Juan Zhang, Yu-Xiang Li, Jian-Qiang Yu

**Affiliations:** 1 School of Nursing, Ningxia Medical University, 1160 Sheng Li Street, Yinchuan, 750004 China; 2 Yinchuan Guolong Hospital, 536 Chang Cheng Road, Xing Qing Area, Yinchuan, 750004 China; 3 Department of Medical Oncology, General Hospital of Ningxia Medical University, Yinchuan, 750004 China; 4 Department of Nursing, Ningxia People’s Hospital, 301 Zheng Yuan Street, Yinchuan, 750004 China; 5 Department of Pharmacology, Pharmaceutical Institute of Ningxia Medical University, 1160 Sheng Li Street, Yinchuan, 750004 China

**Keywords:** Analgesia, Breakthrough pain, Cancer patients, Nitrous oxide

## Abstract

**Background:**

The management of breakthrough pain in cancer patients is always a challenge for medical professions. Occurring in 80% of cancer patients with advanced disease, breakthrough pain significantly decreases both patient’s and caregiver’s quality of life. The aim of this study is to assess the analgesic efficacy of a fixed inhaled nitrous oxide/oxygen mixture for adult cancer patients with breakthrough pain.

**Methods/design:**

This is a randomized, placebo-controlled, double-blind study; it will be conducted in the General Hospital of Ningxia Medical University. The target study subjects are at least 18 years old, and are hospitalized cancer patients who are receiving routine opioids to control cancer-related pain but still experience breakthrough pain. A total of 240 patients will be recruited and randomly allocated between three treatment groups (A, B, C) and a control group (group D) in a ratio of 3:1. All treatment groups (A, B, C) will receive standard pain treatment (oral immediate-release morphine) plus a pre-prepared nitrous oxide/oxygen mixture, and the control group (D) will receive the standard pain treatment plus oxygen. Patients, doctors, nurses, and data collectors are all blind to the experiment. Assessments will be taken before treatment (T0), at 5 min (T1) and 15 min (T2) during treatment, and at 5 min after treatment (T3). The primary endpoint measures will be the percentage of patients whose pain is relieved at T1, T2, and T3. Secondary outcome measures will include the safety of treatment, adverse events, and satisfaction from both health professionals and patients.

**Discussion:**

This study aims to provide an effective and practical intervention for a fast breakthrough pain relief and to improve cancer patients’ quality of life significantly. The Evidence-Based Medicine Working Group claim that a randomized, double-blind, placebo-controlled experimental intervention is the most appropriate design to demonstrate its efficacy, so this study could give a new approach to controlling breakthrough pain episodes.

**Trial registration:**

ChiCTR-INC-16008075. Registered on 8 March 2016.

**Electronic supplementary material:**

The online version of this article (doi:10.1186/s13063-016-1739-9) contains supplementary material, which is available to authorized users.

## Background

In 2015, there were approximately 15 million newly diagnosed cancer cases in the world and about 4.3 million new cases in China alone [1, 2]. Cancer pain is common. The estimated prevalence of cancer-related pain is 30–50% for those who are under chronic pain treatment; over 80% of those with advanced disease suffer moderate to severe pain [3]. Most cancer pain is chronic and caused by the tumor. About 50–90% of patients reported intermittent flaring of pain [4] with a 30–40% rate during the early stages of the disease and 70–90% during advanced stages [5].

Cancer pain is classified as background pain or breakthrough pain according to its temporal characteristics [6, 7]. Background pain, also known as chronic persistent pain, refers to a constant or continuous pain that lasts for more than 12 hours per day [6]. Breakthrough pain is a negative prognostic indicator for pain control. The Association for Palliative Medicine of Great Britain and Ireland task group defines breakthrough pain as a transitory increase in pain intensity that occurs either spontaneously or in relation to a specific predictable or unpredictable trigger despite the use of long-term and around-the-clock analgesics to control background pain. With this definition, breakthrough pain includes both spontaneous (idiopathic) and incidental (precipitated) pain. Spontaneous pain is unpredictable. Incidental pain is somewhat predictable and usually categorized into three sub-types: volitional, non-volitional, and procedural [6–13]. More than 50% of breakthrough pain is reported to be spontaneous [13].

Both inter-individual and intra-individual breakthrough pain varies in terms of timing and severity [6]. A typical breakthrough pain episode is characterized by a rapid onset (median interval of 3 min to peak pain) [14], moderate to severe in intensity (numerical pain rating scale > 4), short duration (median 30–60 minutes) [14], and self-limited (median number of 4 (times) episodes per day with a range of 1-14) [8, 15]. Patients with breakthrough pain have significantly greater pain-related functional impairment [3, 16] and decreased satisfaction with their analgesic therapy [6]; these are correlated with increased suicidal thoughts [17]. The unrelieved breakthrough pain increases patients’ psychological distress and susceptibility to such illnesses as depression and anxiety [3, 18], which put a negative impact on quality of life [3, 6, 19]. It also lays an additional economic burden on the healthcare system because it increases emergency outpatient visits, hospital admissions, and hospital stay [20–22].

The World Health Organization three-step analgesia ladder can manage about 80% of background pain with simple interventions [5, 15]. However, the sudden onset, severity, and short duration of breakthrough pain make its management more difficult [15]. Traditionally, breakthrough pain is managed by varying oral immediate-release opioid at 5–15% [23], 10–20% [24], or one-sixth of the total daily opioid dosage [14]. However, in most cases, the pharmacokinetic and pharmacodynamic profiles of such oral opioids do not align with the rapid nature of breakthrough pain [25–27]. Several studies indicated that the average onset of action for oral opioid (including morphine, oxycodone, and hydromorphone) as rescue medications among hospice patients was greater than 30 min [28, 29]. This long onset of action means that the oral immediate-release opioids are not ideal rescue medications for most breakthrough pain. Studies have evaluated the effectiveness of transmucosal fentanyl formulations for breakthrough pain management [29]. As oral or nasal mucosa allows a more rapid absorption and avoids the first-pass metabolism, transmucosal fentanyl’s onset of action is within 10–15 min after administration [29, 30]. However, some research suggested that the oral transmucosal fentanyl citrate showed a negative correlation with a fixed-schedule opioid regimen and patients have to undergo up to 26 days of the dose-titration phase to determine the optimal dose [31, 32]. Another drawback for this preparation is its high cost. Intravenous morphine is another agent that, at 20% of its basal oral dosage, is a fast, safe, and highly effective option for the relief of breakthrough pain. Its onset of action starts in 3 min and peaks at 10 min [33], but this invasive route of administration and the lack of breakthrough pain management guidelines made this approach less favorable.

A self-administered inhaled nitrous oxide/oxygen mixture is available in pre-prepared cylinders. It has been used in various types of pain management, including labor [34], dental procedures [35], trauma [36], burn dressing [37], surgical procedures, and other medical conditions. The nitrous oxide/oxygen mixture has potent analgesic properties but does not cause loss of consciousness. This gas is safe, noninvasive, and an effective form of pain relief, owing to its low blood/gas solubility ratio, which allows for rapid onset (1–2 min) and short duration of action (35–45 s) after discontinuation [38, 39]. The absorbed gas is also readily excreted unchanged mainly through the lungs [40, 41]. The administration of nitrous oxide/oxygen mixture is easy to control. Its side effects is generally disappear quickly after the termination of exposure to the gas [40, 41].

In mainland China, transmucosal fentanyl preparations are not available and have not been used to control breakthrough pain [data from Chinese Marketed Drugs Database]. According to anesthetists, oral immediate-release opioid is the first choice in controlling breakthrough pain in China. However, with its slow onset of action (20–30 min; peak > 45 min) [42], it delays pain relief and reduces patients’ compliance. Studies by Li et al. showed that a fixed diluted nitrous oxide/oxygen mixture would provide a sufficient analgesic effect during the burn-dressing procedure; patients reported the most satisfaction for this method [17, 37]. However, to our knowledge, there are no studies on the use of nitrous oxide/oxygen mixture to treat breakthrough pain yet. We hypothesize that a nitrous oxide/oxygen mixture can provide the same analgesic effect for cancer patients within breakthrough pain episodes. This is a nurse-led, patient-participate, randomized controlled trial. This article describes the study background, design, treatment administration, and data analysis approach.

## Methods

### Study design

**Fig. 1:**
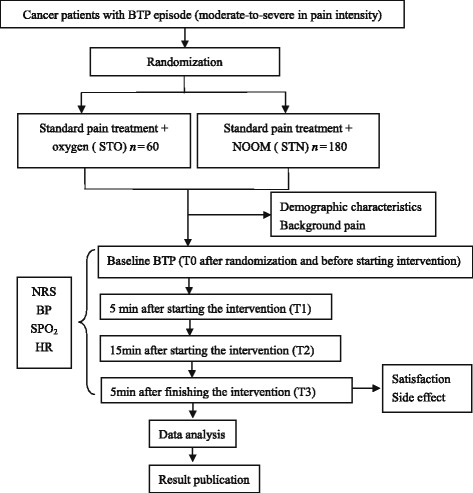
Study design framework. BP, blood pressure; BTP, breakthrough pain; HR, heart rate; NOOM, nitrous oxide/oxygen mixture: NRS, numerical pain rating scale; SPO_2_, oxygen saturation; STN, standard treatment with nitrous oxide/oxygen mixture; STO, standard treatment with oxygen

This study is a randomized, double-blind, placebo-controlled, parallel clinical trial to test the analgesic efficacy of a fixed inhaled nitrous oxide/oxygen mixture on adult cancer patients with breakthrough pain. This article has been drafted following SPIRIT guidelines [43] (see Additional file 1). Patients in the treatment groups will receive standard pain treatment plus pre-prepared nitrous oxide/oxygen mixture and patients in the control group will receive standard pain treatment plus oxygen when they report a breakthrough pain episode. The study protocol will use the Consolidated Standards of Reporting Trials (CONSORT) [44] statement recommendations (see Additional file 2). The overall trial design is provided in Fig. 1. Furthermore, the schedule of enrolment, interventions, and assessments are presented in Table 1. The stratification factors were not contemplated in this study. breakthrough pain is a transitory exacerbation of pain that occurs despite the use of long-term and around-the-clock analgesics to control persistent pain, and there is no nature differences in two types of breakthrough pain. So we did not make stratification analyses in this study.

**Table 1:** Schedule of enrolment, interventions, and assessments

	Study period				
	Enrolment	Post-allocation			Close-out
Timepoint	Diagnosed with breakthrough pain	Breakthrough pain episode (T0)	5 min after starting intervention (T1)	15 min after starting intervention (T2)	5 min after finishing intervention (T3)
Enrolment					
Eligibility screen	√				
Informed consent	√				
Allocation	√				
Interventions					
Control group			√	√	
Treatment groups			√	√	
Assessments					
Numerical pain rating scale		√	√	√	√
Blood pressure		√	√	√	√
Oxygen saturation		√	√	√	√
Heart rate		√	√	√	√
Satisfaction					√
Side effect			√	√	√

### Participants

All cancer patients from the oncology ward who have breakthrough pain will be invited to participate. Davies’ Adapted Diagnostic Algorithm will be used to screen patients for the presence of breakthrough pain [8]. The main inclusion criteria are: Chinese-speaking patients older than 18 years; recent worst pain (breakthrough pain) reported to be 4 or higher on a numerical pain rating scale of 0 to 10; a score on the Karnofsky Performance Status Scale above 20; and ability to take deep breaths in order to use the self-administered device. Patients will be excluded if they have a known contraindication to the use of nitrous oxide or have impaired cognitive function. Specific inclusion and exclusion criteria are presented in Table 2.

**Table 2:** Inclusion and exclusion criteria

Inclusion criteria
Patient aged 18 years or older
Patient experiencing breakthrough pain episode with a stable dose of opioids to control background pain
Written informed consent for participation obtained prior to any study procedures
Exclusion criteria
Patient with mental disorder, altered mental status
Patient has difficulty in reporting pain
Karnofsky Performance Status Scale score under 20
Abdominal distension or suspected bowel obstruction, air embolism, pneumothorax, decompression sickness, epilepsy, pulmonary cancer, chronic obstructive pulmonary disease and acute respiratory infection, pregnancy, severe inhalation injury; pharmaceutical or pathological pulmonary fibrosis; maxillofacial injuries
Disease involving ear, nose, larynges, such as sinuses, middle ear

### Treatments

Eligible patients will receive oral immediate-release morphine with a proportion of 10–20% daily total doses of oral morphine as the standard breakthrough pain treatment [24]. Patients will be instructed to use of study apparatus, which provides a fix concentration of nitrous oxide/oxygen mixture or oxygen to a disposable, demand valve, scented oral–nasal mask. Patients will hold the mask, which is opened by the negative pressure of patients to control the gas taken in. The experimental group will receive a fixed concentration of nitrous oxide/oxygen mixture and the control group will receive oxygen during breakthrough pain episodes. Gas inhalation will be administered until the breakthrough pain episode is over. The project manager is in charge of assignment, allocation, and treatment delivery. All gas cylinders used in both two groups are identical in appearance. The patients, nurse, and data collector are all blind to the treatment.

### Randomization, allocation concealment, and binding

Owing to the severity of the pain, ethicists suggest helping as many suffering patients as possible. In total, 240 participants will be randomized into four groups in a ratio of 3:1; three treatment groups (A, B, C), *n* = 180, and one control group (D), *n* = 60. The allocation sequence of each patient is decided by a computer-generated schedule, which is numbered by a statistician. The randomization schedule will be kept and sealed in an independent research room. Apart from the project manager, who is responsible for gas distribution, no other nurses or data collectors will have access to the data allocation. The nurse is blind to the double-blind randomized controlled trial lists (see Additional file 3) because the list does not indicate what treatment letters A, B, C, and D stand for.

### Measurement

Patient’s demographic data (age, sex, nationality, height, weight), cancer diagnosis, baseline breakthrough pain intensity, current background pain opioid medication dosage, duration of nitrous oxide/oxygen mixture use, dosage of oral rescue morphine medication, and any side or adverse effects of nitrous oxide/oxygen mixture treatment will be recorded by researchers [45]. A numerical pain rating scale (range from 0 to 10) is used to assess for pain intensity along with heart rate, noninvasive monitoring of blood pressure, and digitally monitored oxygen saturation at baseline (T0 before treatment), 5 min (T1) and 15 min (T2) after the beginning of treatment, and at 5 min (T3) after treatment finished. Heart rate, blood pressure, and oxygen saturation will be monitored by Infinity® Delta XL (Draeger Medial Systems Inc., Danvers, Shanghai, China). A five-point satisfaction scale (5, very satisfied; 4, satisfied; 3, uncertain; 2, dissatisfied; 1, very dissatisfied) will be used to assess both patients’ and healthcare professionals’ satisfaction with breakthrough pain treatment [46]. Any side effects of the nitrous oxide/oxygen mixture treatment, such as nausea, vomiting, dizziness, drowsiness, and headache [17], will be carefully monitored by researchers after the initiation of the gases inhalation.

### Endpoints

#### Primary endpoint measures

The primary endpoint measures will be the percentage of patients receiving pain relief at T1, T2, and T3. Repeated-measures analysis of variance will be used for data analysis.

#### Secondary endpoint measures

Secondary outcome measures will include treatment safety, adverse events, and satisfaction from both health professionals and patients. The total time of nitrous oxide/oxygen mixture administration will be also recorded.

### Sample size determination

According to our previous study on burn-dressing pain [17, 37], we used preliminary observational data obtained in medical oncology for 20 patients. We found that 90% of patients receiving pre-prepared nitrous oxide/oxygen mixture experienced pain relief (a decrease of at least 30% in pain intensity [47]) at 15 min versus 10% of patients in the control group. The aim of this study is to assess the efficacy of the nitrous oxide/oxygen mixture, so a sample size of 12 was targeted with a two-tail test with type-1 error rate of 0.05 and a power of 90% to be sufficient. We decided on a sample size of 240 to meet the Chinese Food and Drug Administration standard for feasibility and the safety of nursing staff in implementing this analgesic [17].

### Data management and analysis

Before the study, all data collectors will be trained in the data collection procedure. Data from withdrawn patients will not be used in the final analysis. SPSS version 22.0 (SPSS Inc., IBM Company, Chicago, IL, USA) will be used to conduct quantitative analysis following the intention-to-treat principle for a case-control randomized trial. Descriptive statistics will be analyzed by medians (inter-quartile ranges), means (standard deviations), and proportions (exact binomial 95% confidence intervals), as appropriate. Proportions will be compared by using the chi squared test or the Fisher’s exact test. Means will be compared using Student’s *t* test (normal distribution parameters) or a nonparametric two-sample Mann–Whitney test (non-normal distribution parameters). Statistical significance will be considered for *P* < 0.05.

## Discussion

Many studies on transmucosal fentanyl showed excellent breakthrough pain reduction after 15 min administration [11, 23, 30, 48]. But that means patients have to suffer for at least 15 minutes severe pain. Furthermore, this fentanyl preparation is unavailable in China. In addition to the incompliance and adverse effects of oral immediate-release morphine, many other opioid formulations also appeared incapable of effectively relieving breakthrough pain due to their slow onset of actions (15–60 minutes) [11, 23, 30, 48]. This study intends to find a better alternative rescue medication for cancer patients with breakthrough pain in China.

The study of Li et al. on burns-dressing procedure pain showed faster pain reduction, within 15–20 s, for the pre-prepared nitrous oxide/oxygen mixture group [17, 37]. However, another study found that inhaled nitrous oxide showed a non-significant reduction in pain scores in metastatic dying patients with cancer-related incident pain [49]. In this study, we will attempt to verify both the analgesic efficacy of the fixed nitrous oxide/oxygen mixture, and patients’ and healthcare workers’ satisfaction in using the nitrous oxide/oxygen mixture for breakthrough pain management.

To our knowledge, this study is the first randomized controlled trial to evaluate the effectiveness of the pre-prepared nitrous oxide/oxygen mixture to treat breakthrough pain in cancer patients with advanced disease. If this treatment appears beneficial, this study can help to generate preliminary guidelines on breakthrough pain management in cancer patients with advanced disease. We intend to disseminate the results of this study to international journals and conferences.

### Trial status

Patient recruitment will start on 24 July 2016.

## Additional files

Additional file 1: SPIRIT 2013 checklist: recommended items to address in a clinical trial protocol and related documents. (DOC 122 kb)
Additional file 2: CONSORT 2010 checklist of information to include when reporting a randomized trial. (DOC 218 kb)
Additional file 3: Randomized controlled trial lists for intervention. (DOC 212 kb)
