# Supplementary material for: A fixed inhaled nitrous oxide/oxygen mixture as an analgesic for adult cancer patients with breakthrough pain: study protocol for a randomized controlled trial
Source: Trials. 2017 Jan 11;18:13. doi: 10.1186/s13063-016-1739-9 (PMC5225628; doi:10.1186/s13063-016-1739-9)
Supplement: Additional file 3: — Randomized controlled trial lists for intervention. (DOC 212 kb) [file 13063_2016_1739_MOESM3_ESM.doc]

**Additional files 3 RCT lists for intervention**

| ward | number | intervention |
| --- | --- | --- |
| 3 | 1 | C |
| 3 | 2 | B |
| 3 | 3 | D |
| 3 | 4 | A |
| 3 | 5 | D |
| 3 | 6 | A |
| 3 | 7 | B |
| 3 | 8 | C |
| 3 | 9 | C |
| 3 | 10 | D |
| 3 | 11 | B |
| 3 | 12 | A |
| 3 | 13 | A |
| 3 | 14 | B |
| 3 | 15 | D |
| 3 | 16 | C |
| 3 | 17 | A |
| 3 | 18 | C |
| 3 | 19 | D |
| 3 | 20 | B |
| ward | number | intervention |
| 3 | 21 | D |
| 3 | 22 | A |
| 3 | 23 | C |
| 3 | 24 | B |
| 3 | 25 | D |
| 3 | 26 | B |
| 3 | 27 | A |
| 3 | 28 | C |
| 3 | 29 | B |
| 3 | 30 | C |
| 3 | 31 | A |
| 3 | 32 | D |
| 3 | 33 | D |
| 3 | 34 | C |
| 3 | 35 | A |
| 3 | 36 | B |
| 3 | 37 | A |
| 3 | 38 | D |
| 3 | 39 | B |
| 3 | 40 | C |
| 3 | 41 | A |
| 3 | 42 | C |
| 3 | 43 | D |
| 3 | 44 | B |
| 3 | 45 | C |
| 3 | 46 | B |
| 3 | 47 | A |
| 3 | 48 | D |
| 3 | 49 | D |
| 3 | 50 | C |
| 3 | 51 | A |
| 3 | 52 | B |
| 3 | 53 | C |
| 3 | 54 | B |
| 3 | 55 | A |
| 3 | 56 | D |
| 3 | 57 | B |
| 3 | 58 | C |
| 3 | 59 | A |
| 3 | 60 | D |
| 3 | 61 | C |
| 3 | 62 | D |
| 3 | 63 | B |
| 3 | 64 | A |
| 3 | 65 | B |
| 3 | 66 | A |
| 3 | 67 | C |
| 3 | 68 | D |
| 3 | 69 | B |
| 3 | 70 | C |
| 3 | 71 | D |
| 3 | 72 | A |
| 3 | 73 | B |
| 3 | 74 | A |
| 3 | 75 | C |
| 3 | 76 | D |
| 3 | 77 | C |
| 3 | 78 | B |
| 3 | 79 | D |
| 3 | 80 | A |
| 2 | 81 | B |
| 2 | 82 | D |
| 2 | 83 | C |
| 2 | 84 | A |
| 2 | 85 | A |
| 2 | 86 | C |
| 2 | 87 | D |
| 2 | 88 | B |
| 2 | 89 | D |
| 2 | 90 | B |
| 2 | 91 | C |
| 2 | 92 | A |
| 2 | 93 | D |
| 2 | 94 | B |
| 2 | 95 | A |
| 2 | 96 | C |
| 2 | 97 | B |
| 2 | 98 | C |
| 2 | 99 | A |
| 2 | 100 | D |
| 2 | 101 | C |
| 2 | 102 | B |
| 2 | 103 | A |
| 2 | 104 | D |
| 2 | 105 | B |
| 2 | 106 | A |
| 2 | 107 | C |
| 2 | 108 | D |
| 2 | 109 | C |
| 2 | 110 | A |
| 2 | 111 | B |
| 2 | 112 | D |
| 2 | 113 | C |
| 2 | 114 | B |
| 2 | 115 | D |
| 2 | 116 | A |
| 2 | 117 | B |
| 2 | 118 | A |
| 2 | 119 | D |
| 2 | 120 | C |
| 2 | 121 | A |
| 2 | 122 | C |
| 2 | 123 | B |
| 2 | 124 | D |
| 2 | 125 | D |
| 2 | 126 | C |
| 2 | 127 | B |
| 2 | 128 | A |
| 2 | 129 | A |
| 2 | 130 | B |
| 2 | 131 | C |
| 2 | 132 | D |
| 2 | 133 | B |
| 2 | 134 | D |
| 2 | 135 | A |
| 2 | 136 | C |
| 2 | 137 | A |
| 2 | 138 | B |
| 2 | 139 | C |
| 2 | 140 | D |
| 2 | 141 | D |
| 2 | 142 | A |
| 2 | 143 | C |
| 2 | 144 | B |
| 2 | 145 | C |
| 2 | 146 | B |
| 2 | 147 | D |
| 2 | 148 | A |
| 2 | 149 | A |
| 2 | 150 | C |
| 2 | 151 | D |
| 2 | 152 | B |
| 2 | 153 | A |
| 2 | 154 | D |
| 2 | 155 | C |
| 2 | 156 | B |
| 2 | 157 | C |
| 2 | 158 | B |
| 2 | 159 | D |
| 2 | 160 | A |
| 1 | 161 | B |
| 1 | 162 | D |
| 1 | 163 | C |
| 1 | 164 | A |
| 1 | 165 | C |
| 1 | 166 | A |
| 1 | 167 | D |
| 1 | 168 | B |
| 1 | 169 | D |
| 1 | 170 | A |
| 1 | 171 | C |
| 1 | 172 | B |
| 1 | 173 | A |
| 1 | 174 | C |
| 1 | 175 | B |
| 1 | 176 | D |
| 1 | 177 | C |
| 1 | 178 | B |
| 1 | 179 | A |
| 1 | 180 | D |
| 1 | 181 | B |
| 1 | 182 | A |
| 1 | 183 | D |
| 1 | 184 | C |
| 1 | 185 | B |
| 1 | 186 | D |
| 1 | 187 | A |
| 1 | 188 | C |
| 1 | 189 | C |
| 1 | 190 | B |
| 1 | 191 | D |
| 1 | 192 | A |
| 1 | 193 | A |
| 1 | 194 | B |
| 1 | 195 | C |
| 1 | 196 | D |
| 1 | 197 | A |
| 1 | 198 | C |
| 1 | 199 | B |
| 1 | 200 | D |
| 1 | 201 | C |
| 1 | 202 | A |
| 1 | 203 | B |
| 1 | 204 | D |
| 1 | 205 | A |
| 1 | 206 | C |
| 1 | 207 | D |
| 1 | 208 | B |
| 1 | 209 | D |
| 1 | 210 | B |
| 1 | 211 | A |
| 1 | 212 | C |
| 1 | 213 | D |
| 1 | 214 | B |
| 1 | 215 | A |
| 1 | 216 | C |
| 1 | 217 | B |
| 1 | 218 | C |
| 1 | 219 | D |
| 1 | 220 | A |
| 1 | 221 | D |
| 1 | 222 | C |
| 1 | 223 | B |
| 1 | 224 | A |
| 1 | 225 | A |
| 1 | 226 | B |
| 1 | 227 | C |
| 1 | 228 | D |
| 1 | 229 | A |
| 1 | 230 | D |
| 1 | 231 | C |
| 1 | 232 | B |
| 1 | 233 | D |
| 1 | 234 | A |
| 1 | 235 | C |
| 1 | 236 | B |
| 1 | 237 | A |
| 1 | 238 | D |
| 1 | 239 | B |
| 1 | 240 | C |
